# Amyloid in biopsies of the gastrointestinal tract—a retrospective observational study on 542 patients

**DOI:** 10.1007/s00428-016-1916-y

**Published:** 2016-02-25

**Authors:** Sophie Freudenthaler, Ute Hegenbart, Stefan Schönland, Hans-Michael Behrens, Sandra Krüger, Christoph Röcken

**Affiliations:** Department of Pathology, Christian-Albrechts-University, Arnold-Heller-Str. 3, Haus 14, 24105 Kiel, Germany; Medical Department V, Amyloidosis Center, University of Heidelberg, Heidelberg, Germany

**Keywords:** Amyloid, Gastrointestinal tract, Biopsy

## Abstract

In this retrospective observational study, we investigated the histopathological and demographic characteristics of amyloid in gastrointestinal biopsies. From the Amyloid Registry Kiel, we retrieved all cases with amyloid in biopsies of the stomach, duodenum, small intestine, large intestine, and rectum submitted for tertiary referral between January 2003 and April 2013. Amyloid was identified by Congo red staining in combination with polarization microscopy and classified by immunohistochemistry. The *TTR*-genotype was assessed in 56 patients. Amyloid type was correlated with demographic patient characteristics. Six hundred sixty-three biopsies from 542 patients were retrieved. Amyloid was found in each biopsy as vascular and/or interstitial amyloid deposits. Biopsies were obtained from the colon [254 biopsies (38.3 %)], stomach, [153 (23.1 %)], rectum [112 (16.9 %)], duodenum [105 (15.8 %)], and jejunum/ileum [39 (5.9 %)]. ALλ amyloid was found in 286 (52.8 %), ATTR in 88 (16.2 %), ALκ in 74 (13.7 %), AA in 58 (10.7 %), and ApoAI amyloid in 4 (0.7 %) patients. The remaining 21 cases were ALys amyloid in 4 (0.7 %), AL n.o.s. in 14 (2.6 %), and mixed type amyloidosis in 3 (0.6 %). The amyloid of 11 (2.0 %) cases remained unclassified. The median age of the patients was 68 years. Men [332 (61.7 %)] were significantly more prevalent than women [206 (38.3 %); *p* < 0.001]. *TTR* mutations were found in 24 % of the patients with ATTR amyloidosis. The median age, the histoanatomical distribution (proximal to distal; mucosal to submucosal), and the deposition pattern (vascular/interstitial) varied between different amyloid types. Amyloid in gastrointestinal biopsies mainly affects male elderly patients and shows amyloid-type-specific demographic patient characteristics.

**Electronic supplementary material:**

The online version of this article (doi:10.1007/s00428-016-1916-y) contains supplementary material, which is available to authorized users.

## Introduction

Amyloid is characterized by the pathological deposition of peptides and proteins in diverse tissues and organs interfering with normal tissue and organ function. It consists of misfolded, insoluble, toxic peptide aggregates, which are oriented in a β-sheet structure [1]. Up to now, 31 autologous, physiological proteins have been identified, which can form amyloid locally or systemically in diverse tissues and organs [2]. The diagnosis is based on the histological examination of an amyloid-containing specimen and the detection of a characteristic green-yellow-orange birefringence under polarized light after Congo red staining [3]. Nearly every organ or tissue type can be affected, with the kidney, liver, heart, tongue, autonomic nervous system, and gastrointestinal tract being among the most common, clinically relevant, extracerebral anatomical sites [3–5]. Renal amyloidosis can cause proteinuria and renal failure [6–8]. Cardiac amyloidosis can lead to arrhythmias, congestive heart failure, and patient death [9–11]. Gastrointestinal amyloidosis can be associated with abdominal pain, nausea, diarrhea, obstipation, and weight loss [5, 12].

Different types of amyloid may show different clinical pictures. The most common type, i.e., immunoglobulin light chain-derived (AL-) amyloidosis, can occur as local or systemic variant and is able to involve almost every organ/tissue type [13, 14]. However, renal and cardiac involvement is found in more than 50 % of the patients [1]. Transthyretin-derived (ATTR-) amyloidosis occurs as hereditary form due to a point mutation in the *TTR* gene or as wildtype variant without a germline mutation. Clinical presentation is characterized by two main manifestations including senso-motoric polyneuropathy and (restrictive) cardiomyopathy [15–17]. Amyloid A (AA-) amyloidosis mainly presents with renal involvement [1]. Hereditary apolipoprotein AI-derived (AApoAI-) is a systemic disease and frequently involves the liver, kidney, larynx, skin, and myocardium [18]. Clinical presentation of hereditary lysozyme-derived (ALys-) amyloidosis is variable and may present with renal manifestations, gastrointestinal symptoms, and bleeding events [19, 20]. Amyloidosis can be treated and therapy depends on early diagnosis and a correct classification [3].

Almost 60 years ago, rectal biopsy was introduced as a diagnostic procedure for the detection of amyloid [21, 22]. Since then, our knowledge of the pathology of amyloid and amyloidosis as well as diagnostic tools (e.g., flexible endoscopy) used by gastroenterologists improved substantially and it is well recognized that amyloid can affect diverse sites of the gastrointestinal tract, leading to the conjecture that rectal biopsy may not be the only location suitable for the detection of amyloid. In addition, we have learnt that the diverse forms of amyloid show unique patterns of organ manifestation.

In this retrospective observational study on the hitherto largest series of amyloid- containing biopsies obtained from the gastrointestinal tract, we tested the following hypotheses: (1) the gastrointestinal tract is affected by diverse types of amyloid, the different types of amyloid show (2) specific demographic patient characteristics and (3) unique proximal-distal (horizontal) and mucosal-submucosal (vertical) distribution patterns, and (4) hereditary ATTR amyloidosis can affect the gastrointestinal tract.

## Material and Methods

### Patients

From the Amyloid Registry Kiel, we retrieved all cases with histologically proven amyloid in biopsy specimens of the stomach, duodenum, small intestine, large intestine, and rectum. Esophageal biopsies were not included, as only six biopsies were documented in the Amyloid Registry. A biopsy is defined as a collection of biopsy fragments obtained from a given site in a given patient at a given time point. All biopsy specimens were obtained between January 2003 and April 2013 and referred to the Amyloid Registry for a second opinion, i.e., confirmation of amyloid, and subsequent classification of the amyloid type. Forty eight biopsies from 45 patients submitted to the Amyloid Registry Kiel were excluded from this series. The presence of amyloid could not be confirmed. Clinical information was not available, as almost all referrals were submitted by surgical pathologists after they had reached a diagnosis of amyloid in the tissue specimens. This study was performed according to the Declaration of Helsinki. Ethical approval was obtained from the local ethical review board (D 581/15-585/15). All patient data were pseudonymized prior to study inclusion. Written informed consent was not sought for this retrospective observational study on archival tissue specimens. Samples were anonymized prior to non-individualizing *TTR*-gene testing.

### Histology

All tissue biopsies had been fixed in formalin and embedded in paraffin. At the Amyloid Registry, serial sections were cut from each paraffin block and stained with hematoxylin and eosin, Congo red, and for immunostaining (see below). The presence of amyloid was confirmed when a typical green-yellow-orange birefringence was found in cross-polarized light in Congo red-stained sections. The anatomical distribution of amyloid was documented for every biopsy specimen with regard to vascular and interstitial as well as mucosa, muscularis mucosae, and submucosa.

### Immunohistochemistry

The immunohistochemistry was carried out with commercially available monoclonal antibodies directed against AA amyloid (1:2000) and polyclonal antibodies directed against amyloid P-component (1:2000), fibrinogen (1:1000), lysozyme (1:2000), prealbumin (1:3000), lambda-light chain (1:1:50,000), and kappa-light chain (1:100,000; all DAKO, Hamburg Germany) and non-commercially available polyclonal antibodies directed against apolipoprotein A1 (anti-apo A1; dilution 1:1000), lambda-light chain-derived amyloid proteins (AL1 antibody, 1:250), anti-lambda-light chain peptide antibodies (AL3, 1:250; AL7, 1:200), transthyretin (TTR3, 1:2000), and kappa-light chain amyloid proteins (AK3, 1:1000). Immunostaining was done on formalin-fixed and paraffin-embedded sections with the BenchMark®XT immunostainer using the ultraView™ Universal Alkaline Phosphatase Red (in older cases brown) Detection Kit (both from Ventana Medical Systems, Inc. Tucson, Arizona) or with the Bond Max Leica immunostainer using the Bond Polymer Refine Red Detection Kit (Leica Microsystems, Wetzlar, Germany). Antigen retrieval was carried out with ER2-Bond Epitope Retrieval Solution 2 (amyloid P-component, lambda-light chain, kappa-light chain, TTR3, and prealbumin), ER1-Bond Epitope Retrieval Solution 1 (apo A1 and fibrinogen), or Enzyme 1 (AL7; all Leica Microsystems, Germany) according to the manufacturer’s instructions. Immunohistochemical classification was carried out and had been validated as described in detail elsewhere [23–27]. In brief, identification of the amyloid was considered to be positive when there was a strong and homogenous immunostaining of the entire amyloid deposits. Uneven and weak staining of some deposits was not assumed to be proof of the amyloid protein. If the staining was clearly positive with more than one antibody against different amyloid precursor proteins, the case was categorized as mixed amyloidosis. AL amyloid not otherwise specified (n.o.s.) were characterized by staining with antibodies directed against λ- and κ-light chain.

Immunostaining with antibodies directed against fibrinogen and lysozyme were done routinely until 2011. Subsequently, immunostaining was done with these antibodies only when AFib- or ALys-amyloidosis was within the differential diagnosis. The anti-prealbumin antibody was replaced in 2010 by the anti-TTR peptide antibody (TTR3). The anti-lambda light chain antibody AL7 was introduced in 2007, while the antibody directed against AL3 was used until 2011. The anti-kappa-light chain antibody AK3 was routinely used since 2011. On slide positive and negative controls using a tissue microarray with AA-, ALλ-, and ATTR-amyloid as well as non-neoplastic liver tissue were used on each staining round.

### Assessment of TTR-genotype

The *TTR*-genotype (exons 1 through 4) was assessed as described in detail previously using formalin-fixed and paraffin-embedded tissue samples. The numbering of amino acid residues refers to the mature TTR protein following the guidelines of the International Society of Amyloidosis [2].

### Statistics

Analyses and statistical tests were carried out with IBM SPSS Statistics Version 20. Significances of correspondence between variables in cross tables were determined using Fisher’s exact test. Significances of differences between age distributions of the amyloidosis types were tested using Kruskal-Wallis test (overall differences) and Mann-Whitney *U* test (pairwise differences). Chi-square test for equal distribution was used to test distributions of localization for the distinct amyloidosis types. Significance of differences between proportions was tested using the “2-sample test for equality of proportions with continuity correction” from R Version 3.2.0. All *p*-values are given unadjusted. A *p* ≤ 0.05 was considered statistically significant. Effects of multiple testing were accounted for by group-wise application of the Simes (Benjamini-Hochberg) procedure for control of false discovery rate [28].

## Results

Six hundred sixty-three biopsies from 542 patients were available with amyloid. AL amyloid of lambda light chain origin (ALλ) was found in 286 (52.8 %), ATTR amyloid in 88 (16.2 %), AL amyloid of kappa-light chain origin (ALκ) in 74 (13.7 %), and AA amyloid in 58 (10.7 %) cases. ApoAI amyloid was an uncommon diagnosis in biopsies of the gastrointestinal tract [4 (0.7 %) cases] as well as ALys amyloid [4 (0.7 %)]. In 14 (2.6 %) patients with AL amyloidosis, the subclassification of amyloid was impossible and the cases were categorized as AL n.o.s. amyloidosis. Mixed amyloid showing clearly positive staining with more than one antibody against different amyloid precursor proteins was found in biopsies obtained from 3 (0.6 %) patients, i.e., AA- and ATTR-amyloid, ALλ- and ATTR-amyloid, and ALκ- and ATTR each in a single colon or rectal biopsy. In 11 (2.0 %) cases, the amyloid deposits remained unclassified.

Biopsies from two different anatomical regions were available from 86 patients, three from 16 patients and four from a single patient. In all cases with biopsies obtained from different anatomical regions of the gastrointestinal tract, the amyloid type classified by immunohistochemistry was identical.

### Patient demographics

First, we correlated the distribution of the different types of amyloid with patient age and gender.

The overall median age at diagnosis was 68.0 years (range 17 to 100 years; Table 1). In five patients, the age was unknown. The highest median age was found in ATTR amyloidosis (73.0 years), followed by ALκ- (68.0 years), ALλ- (66.0 years), and AA amyloidosis (64.0 years). Cases with unclassified amyloid deposits had the lowest median age (61.0 years). Testing the distribution of all medians together for randomization, a *p* value <0.001 was calculated, indicating that the median age distribution of the different types of amyloidosis is not random. Subsequently, we compared the amyloid types directly. The difference in median age was found to be significant between ALλ- and ATTR amyloidosis and between AA- and ATTR amyloidosis (*p* < 0.001; respectively). Interestingly, no statistical difference was found between unclassified amyloidosis (61.0 years) and ALλ- or ALκ amyloidosis.

**Table 1:** Correlation of amyloid types with patient age and gender

Amyloid-type	Age at diagnosis			Age groups [*n* (%)]								Gender [*n* (%)]		
	Total [*n* (%)]	Median [years]	Range [years]	<31	31–40	41–50	51–60	61–70	71–80	81–90	>90	Total	Male	Female
AL lambda	283 (52.7)	66.0	25–100	1 (0.4)	2 (0.7)	23 (8.1)	52 (18.4)	97 (34.3)	95 (33.6)	12 (4.2)	1 (0.4)	283 (52.6)	184 (65.0)	99 (35.0)
AL kappa	74 (13.8)	68.0	45–93	0 (0.0)	0 (0.0)	4 (5.4)	13 (17.6)	22 (29.7)	28 (37.8)	6 (8.1)	1 (1.4)	74 (13.8)	39 (52.7)	35 (47.3)
AL n.o.s.	13 (2.4)	69.0	40–86	0 (0.0)	1 (7.7)	0 (0.0)	1 (7.7)	5 (38.5)	3 (23.1)	3 (23.1)	0 (0.0)	14 (2.6)	9 (64.3)	5 (35.7)
ATTR	88 (16.4)	73.0	40–92	0 (0.0)	0 (0.0)	5 (5.7)	12 (13.6)	12 (13.6)	37 (42.0)	21 (23.9)	1 (1.1)	88 (16.4)	53 (60.2)	35 (39.8)
AA	57 (10.6)	64.0	32–86	0 (0.0)	5 (8.8)	3 (5.3)	13 (22.8)	18 (31.6)	14 (24.6)	4 (7.0)	0 (0.0)	57 (10.6)	33 (57.9)	24 (42.1)
AApoAI	4 (0.7)	67.5	62–75	0 (0.0)	0 (0.0)	0 (0.0)	0 (0.0)	3 (75.0)	1 (25.0)	0 (0.0)	0 (0.0)	4 (0.7)	2 (50.0)	2 (50.0)
ALys	4 (0.7)	41.5	17–65	1 (25.0)	1 (25.0)	1 (25.0)	0 (0.0)	1 (25.0)	0 (0.0)	0 (0.0)	0 (0.0)	4 (0.7)	2 (50.0)	2 (50.0)
Mixed	3 (0.6)	72.0	71–72	0 (0.0)	0 (0.0)	0 (0.0)	0 (0.0)	0 (0.0)	3 (100.0)	0 (0.0)	0 (0.0)	3 (0.6)	1 (33.3)	2 (66.7)
unclassified	11 (2.0)	61.0	44–87	0 (0.0)	0 (0.0)	3 (27.3)	1 (9.1)	3 (27.3)	2 (18.2)	2 (18.2)	0 (0.0)	11 (2.0)	9 (81.8)	2 (18.2)
Total	537^a^ (100.0)	68.0	17–100	2 (0.4)	8 (1.5)	40 (7.4)	92 (17.1)	161 (30.0)	183 (34.1)	48 (8.9)	3 (0.6)	538^b^ (100.0)	332 (61.7)	206 (38.3)

^a^In 5 patients, the age was not known

^b^In 4 patients, the gender was not known

In general, amyloid in the gastrointestinal tract seems to be a finding in the elderly, since over 70 % of the patients were ≥60 years of age (Table 1). The majority (67 %) of patients with ATTR amyloidosis were even >70 years old. An exception to this rule was found in AA amyloidosis. Thirty seven percent of the patients with AA amyloidosis were younger than 61 years.

Three hundred thirty-two (61.7 %) patients were male and 206 (38.3 %) female. In four (0.7 %) cases, the gender was unknown (Table 1). The gender difference was significant (*p* < 0.001) irrespective of the amyloid type. However, few non-significant variations were noticed in gender ratio. In ALλ, the ratio (men vs. women) was 65.0 vs. 35.0 % and in ALκ 52.7 vs. 47.3 %. In four patients with AApoAI amyloid, the gender ratio was even, and in 11 patients with unclassified amyloid, the ratio was 81.8 vs. 18.2 %

### Histoanatomical distribution of amyloid

Next, we assessed the distribution pattern of the different types of amyloid from proximal to distal (“horizontal”) and from the lamina propria to the submucosa (“vertical”).

#### Anatomical distribution from proximal to distal (“horizontal”)

**Fig. 1:**
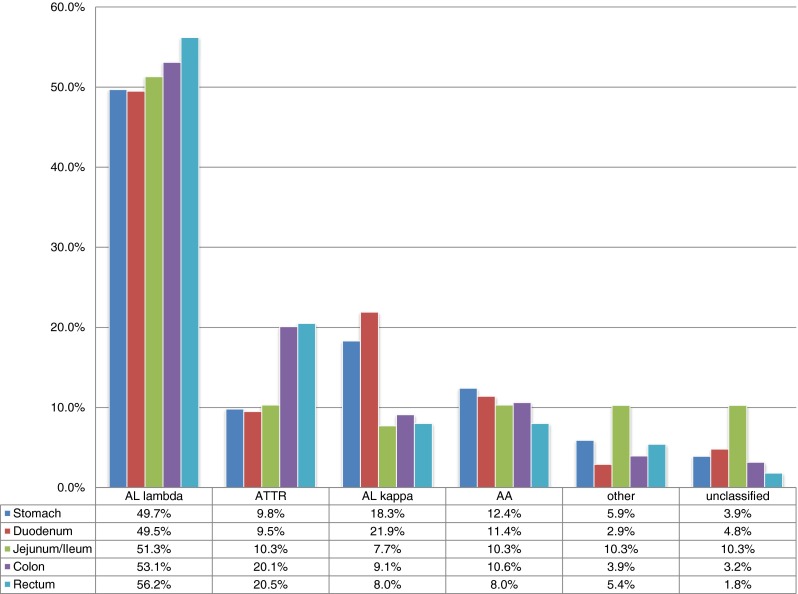
Proportional prevalences of different amyloid types in gastrointestinal biopsies: anatomical distribution from proximal to distal

Most biopsies were obtained from the colon [254 biopsies (38.3 %)], followed by the stomach, [153 (23.1 %)], rectum [112 (16.9 %)], duodenum [105 (15.8 %)], and jejunum/ileum [=small intestine; 39 (5.9 %)]. With regard to total numbers, each type of amyloid was found most commonly in colon biopsies, except for ALκ amyloidosis, which was found more often in stomach biopsies [stomach 29 (31.2 %); colon 26 (28.0 %)] and also unclassified amyloidosis [stomach 5 (33.3 %); colon 3 (20.0 %)]. Since total numbers might be biased by preferred sampling sites, we next analyzed the proportional distribution of the different amyloid types (Fig. 1). This showed that ALλ amyloidosis was the most common amyloid type in every anatomical region of the gastrointestinal tract (on average 52.3 %). Interestingly, its proportional prevalence steadily increased from 49.7 % in the stomach to 56.2 % in the rectum (Fig. 1). The second most common type was ATTR amyloid (on average 15.7 %), showing a similar increase in its proportional prevalence from stomach (9.8 %) to rectal biopsy sites (20.5 %; Fig. 1). An inverse proportional prevalence was found for ALκ- and AA amyloidosis (Fig. 1). ALκ- and AA amyloidosis were found most commonly in stomach and duodenal biopsies. Unclassified amyloidoses were most prevalent in biopsies of the small intestine.

The “horizontal” proportional distribution of the different amyloid types was statistically significant with regard to the entire group (*p* = 0.003) as well as with regard to ALκ- (*p* = 0.001) and ATTR amyloidosis (*p* = 0.006). However, the uneven horizontal distribution of ALλ- (*p* = 0.805) and AA amyloidosis (*p* = 0.849) was insignificant. The number of biopsies with other types of amyloid and unclassifiable amyloid were too small for statistical analyses.

#### Anatomical distribution from the lamina propria to the submucosa (“vertical”)

**Fig. 2:**
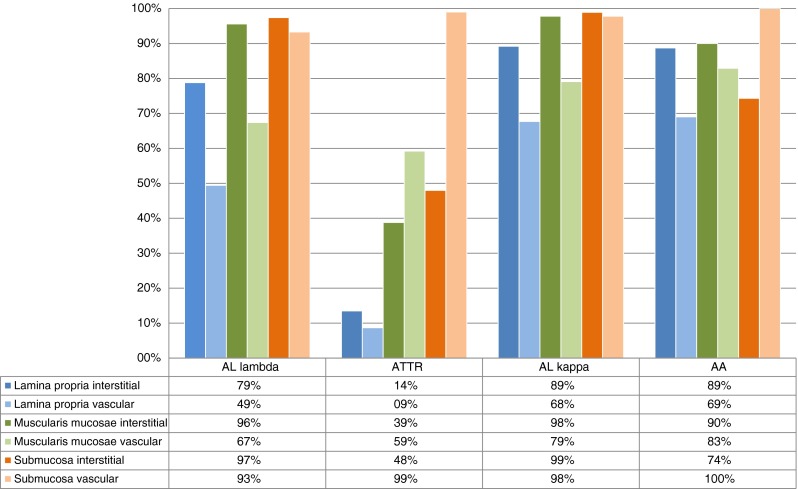
Proportional prevalences of different amyloid types in gastrointestinal biopsies: anatomical distribution from mucosal to submucosal

Next, we analyzed the proportional distribution of the different amyloid types vertically, i.e., proportional distribution of the different amyloid types in the lamina propria, muscularis mucosae, and submucosa irrespective of the horizontal distribution (Fig. 2; Suppl. Table 1).

Six hundred sixty (99.5 %) of 663 biopsies included lamina propria, amyloid was found in 477 (72.3 %) biopsies. Involvement of the lamina propria was most prevalent in the stomach [132 biopsies (86.3 %)] followed by duodenum [79 (75.2 %)], jejunum/ileum [=small intestine; 25 (64.1 %)], colon [170 (67.2 %)], and rectum [71 (64.5 %)]. The differences between stomach and duodenum (*p* = 0.032), as well as stomach and colon (*p* < 0.001), were statistically significant. However, after correction for multiple testing, the difference between stomach and duodenum lost significance (adjusted *p* limit <0.031). Amyloid deposits in the lamina propria were found more commonly in the interstitium [472 (99 %) biopsies] and less commonly in vessel walls [314 (65.8 %)]. Interstitial deposits only were found in 163 (34.2 %) biopsies, a mixed interstitial/vascular deposition pattern in 309 (64.8 %) biopsies, and vascular involvement only in 5 (1 %; *p* < 0.001). Irrespective of the amyloid type, amyloidosis of the lamina propria was most prevalent in the stomach compared with more aborally located biopsy sites

**Fig. 3:**
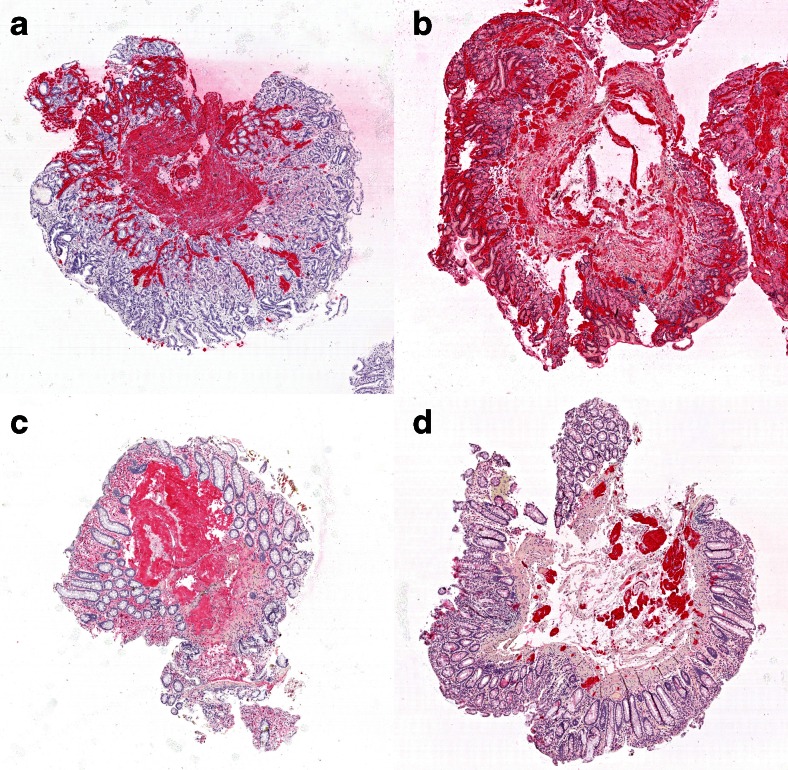
Comparison of the deposition pattern of four with different types of amyloid. AA-amyloid in a stomach biopsy (**a**), ALλ amyloid in a stomach biopsy (**b**), ALκ amyloid in a colon biopsy (**c**), and ATTR amyloikd in a rectal biopsy (**d**). Immunostaining with antibodies directed against AA amyloid (**a**), λ-light chain (**b**), κ-light chain (**c**), and transthyretin (**d**). Original magnifications threefold

Among the different types, involvement of the lamina propria was most prevalent in AA amyloidosis [64 (90.1 %)], followed by ALκ- [83 (89.2 %)] and ALλ amyloidosis [273 (79.4 %)]. Involvement was least common in ATTR amyloidosis [16 (15.4 %)] (Fig. 2). The differences between ALλ- and AA amyloidosis did not reach significance after multiple testing (*p* = 0.044; adjusted *p* limit <0.035). However, the difference between ALλ- and ATTR amyloidosis was statistically significant (*p* < 0.001). In addition to the differences in the proportional distribution of the different amyloid types, we also noted differences in the general histological appearance of the amyloid types. In ALλ amyloidosis, involvement of the lamina propria often exhibited small amyloid deposits located in form of a rim under the surface epithelium, while in AA- and ALκ amyloidosis, the amyloid deposits were often gross/patchy and located in the entire mucosa (Fig. 3).

Six hundred fifty-five (98.8 %) of 663 biopsies included muscularis mucosae (Suppl. Table 1). Most of the cases showed amyloid deposits in the interstitium (Fig. 2). The muscularis mucosae was involved in >90 % of the biopsies with ALλ-, ALκ-, or AA amyloidosis (Suppl. Table 1). Vascular amyloid deposits were present in 232 (67.4 %) cases with ALλ amyloidosis, followed by ALκ- [72 (79.1 %)] and AA amyloidosis [58 (82.9 %)]. The difference between ALλ- and AA amyloidosis was significant (*p* = 0.01). In ATTR amyloidosis vascular amyloid deposits of the muscularis mucosae were more prevalent [61 biopsies (59.2 %)] compared with interstitial deposits [40 (38.8 %)]. The difference of interstitial amyloid was significant between ALλ- and ATTR amyloidosis (*p* < 0.001)

649 biopsies enclosed submucosa (Suppl. Table 1). Submucosal vessels were enclosed in 648 biopsies. Every biopsy showed amyloid in the submucosa. Vascular amyloid deposits were found in >90 % of the biopsies. However, differences were found in the proportional distribution among different amyloid types. Interstitial amyloid deposits were found only in 49 biopsies (48.0 %) with ATTR amyloid. Interstitial amyloid deposits were more prevalent in AA- [52 (74.3 %)], ALλ- [333 (97.4 %)], and ALκ amyloidosis [88 (98.9 %)](Fig. 2). The differences between ALλ- and AA- or ATTR- amyloidosis, as well as between ALκ- and AA amyloidosis, and between ATTR- and AA amyloidosis were significant (*p* ≤ 0.001; respectively).

### TTR-genotype

In 56 patients with ATTR amyloidosis, formalin-fixed and paraffin-embedded tissue samples were available for molecular testing. In 18 patients, the quality of the DNA extracted was insufficient, and 38 patients remained, in whom all four exons of the *TTR*-gene could be analyzed. Twenty-nine patients (76.3 %) showed a wild-type sequence and were classified as senile cardiovascular ATTR amyloidosis. Nine (23.7 %) patients carried a mutation and were classified as hereditary ATTR amyloidosis. Patients with hereditary ATTR amyloidosis were significantly younger [median age 70 years; range 43–75 years] compared with those suffering from senile cardiovascular ATTR amyloidosis [median age 77 years; range 42–92 years; *p* = 0.021]. Of nine patients carrying a mutation, 6 were male and 3 female.

### Unclassified Amyloidoses

In the 15 biopsies of 11 patients, the amyloid deposits remained unclassified. The amyloid type of two patients (three biopsies) could not be defined because of technical reasons, i.e., missing serial sections for immunostaining. In the remaining 12 biopsies of 9 patients, the amyloid deposits did not stain with any of our antibodies.

## Discussion

Annually, the Amyloid Registry Kiel receives on average 110 referrals for the classification of amyloid in gastrointestinal biopsy specimens. These represent 21 % of all registry cases and are the most common biopsy site, followed by the heart (18 %) [4] and carpal tunnel ligament (8 %) [23]. The unique centralized service offers the chance to collect large patient series and testing histopathological and demographic characteristics of the diverse types of amyloid. This may improve daily practice in clinics as well as in diagnostic surgical pathology of an otherwise rare disease. Our retrospective observational study on the hitherto largest series of amyloid-bearing gastrointestinal biopsies shows that the gastrointestinal tract is affected by diverse types of amyloid and at different anatomical sites. Any amyloid-containing biopsy of any anatomical site of the gastrointestinal tract is suitable to reach a proper diagnosis and to classify amyloid. However, the different types of amyloid vary with regard to age, gender, vascular-interstitial, proximal-distal (horizontal), and mucosal-submucosal (vertical) distribution patterns. This knowledge may be used to guide biopsy procedures and support classification of amyloid and is therefore of immediate diagnostic value. Previously, it was shown that cardiac biopsies frequently enclose either ALλ- or ATTR amyloid, while liver biopsies comprise a high proportion of ALκ amyloid [4, 29]. These observational findings are now extended to biopsies of the gastrointestinal tract. While ALλ amyloidosis is the most common type in gastrointestinal biopsies, its proportional prevalence increases from proximal to distal, as it also does for ATTR amyloidosis. ALκ amyloidosis shows the opposite relationship. Similarly, the “vertical” (mucosal to submucosal) and vascular/interstitial distribution pattern of the different types of amyloid varies (Fig. 2). Mucosal involvement was common in AA- and ALκ amyloidosis, less frequently in ALλ amyloidosis, and very rare in ATTR amyloidosis. These observations confirm findings made by Said et al. [12]. In their series of 79 cases with gastric amyloidosis, involvement of the lamina propria was observed less frequently in ATTR amyloidosis compared with other types. Mucosal involvement in AA- and less frequently in AL amyloidosis was also reported by others [30–32]. Alcarde-Vargas et al. [33] described differences in the histoanatomical distribution of amyloid between submucosa and lamina propria, which correlated with the endoscopic findings but not with the amyloid type. However, Alcarde-Vargas et al. [33] studied only 24 patients with three different types of amyloid. We found commonly AA-, ALλ-, and ALκ amyloid in the muscularis mucosae (each ≥90 % of the biopsies). Again, interstitial ATTR amyloidosis occurred significantly less frequently in the muscularis mucosae (<40 %), but vascular involvement was encountered more frequently (approx. 60 %). Said et al. [12] and Gilat et al. [34] reported similar findings in AL amyloidosis, while Röcken et al. [26] previously reported lower prevalences (37 %). However, our previous investigation did not correlate the amyloid type with the histoanatomical distribution and enclosed a much lower number of cases with AL amyloidosis [26].

Kyle et al. [22] already reported 50 years ago that tissue sampling influences the sensitivity of amyloid detection being higher when submucosal layers are enclosed. Gastrointestinal biopsies from patients suffering from amyloidosis, which do not contain submucosa, may miss the deposits in more than 60 % [26]. As shown here, the problem of a sampling error is most evident in ATTR amyloidosis. Compared with all other types, mucosal involvement was least common in ATTR amyloidosis (Suppl. Table 1). Submucosal involvement, mostly of vessel walls, was found in every biopsy and has also been reported previously [32, 34, 35]. Thus, ATTR amyloidosis carries the highest risk of a sampling error, when submucosal layers are not enclosed in the biopsy specimen.

Along the proximal-distal axis of the gastrointestinal tract, the anatomical site may also impact on the mucosa/submucosal and vascular/interstitial deposition pattern. Gilat et al. [34] found mucosal involvement of the stomach and duodenum more frequently than in the colon in their series of 68 patients, with only 16 suffering from AL amyloidosis. Yamada et al. [32] reported a higher prevalence of mucosal involvement in AL amyloidosis of the stomach compared with colon on their series of 21 autopsy cases (18 AL- and 3 AA amyloidoses). However, no difference was found for AA amyloidosis. Similarly, in our series, the difference between stomach and colon mucosa was less prominent for AA amyloidosis compared with ALλ amyloidosis. The organ site-specific differences in the deposition pattern of AL amyloidosis prompted Yamada et al. [32] to recommend stomach biopsies as preferable anatomical site for biopsy confirmation of AL amyloidosis: mucosal involvement is much more prevalent in gastric biopsies and limits the risk of sampling bias, when submucosal layers are missed by the biopsy procedure. We would extend this recommendation in stating that ALκ- and AA amyloidosis should be sought in biopsies of the upper gastrointestinal tract, while ALλ- and ATTR amyloidosis should be sought preferentially in colorectal biopsies enclosing submucosal layers.

In Western countries, AL amyloidosis is the most common variant of systemic amyloidosis [1, 5]. As shown here and previously by others, this also applies to the gastrointestinal tract [12, 32, 36, 37], with ALλ being more prevalent than ALκ [12, 32]. However, hereditary amyloidosis may also affect the gastrointestinal tract. We found cases with AApoAI- and ALys amyloidosis. Thus, special care should be taken not to miss hereditary amyloidosis in biopsies of the gastrointestinal tract [38]. Hereditary ATTR amyloidosis is the most prevalent type in Germany, with many patients suffering from gastrointestinal symptoms, some of which may be related to visceral polyneuropathy [39, 40]. Separation of hereditary from wild-type ATTR amyloidosis has clinical implications with regard to therapy and further genetic counseling [41]. In the literature, the prevalence ranges from 24 to 85 % [4, 12, 36, 42]. The differences in the prevalence might be related to patient selection and origin of the biopsy specimen. Higher prevalences are to be expected in endemic regions of hereditary ATTR amyloidosis such as Portugal, Sweden, and Japan [43–48]. Collectively, these data show that hereditary ATTR amyloidosis is sampled by gastrointestinal biopsies and that the median age of these patients (70 years) is in the range of AA- and AL amyloidosis. Additionally, we recommend that genetic counseling should be offered to every patient with ATTR amyloidosis in gastrointestinal biopsies, since patient age shows a considerable overlap between senile systemic and hereditary ATTR amyloidosis and is of little help to distinguish both types.

In summary, amyloid in gastrointestinal biopsies is found preferentially in elderly male patients and is most commonly of AL type. The different types of amyloid show distinctive deposition patterns, which may help to improve diagnostic procedures, i.e. choice of endoscopic biopsy site, and tissue-based classification of amyloid. Based on our findings, we propose that if systemic amyloidosis is suspected clinically, ALκ- and AA amyloidosis should be sought preferentially in biopsies of the upper gastrointestinal tract and ALλ- and ATTR amyloidosis in biopsies of the large intestine enclosing submucosal layers.

## Electronic Supplementary Material

ESM 1 (DOC 77 kb)

Supplemental Figure 1 ALλ amyloid in a gastric biopsy with typical involvement of the muscularis mucosae. On an H&E-stained section (A) a homogeneous eosinophilic material was found in the interstitium extending into the mucosa. Congo red stains the deposits (B) and shows yellow-green-orange birefringence (not shown). The amyloid deposits immunoreact with an antibody directed against lambda-light chain (C) but not with an antibody directed against transthyretin (D). Original magnifications 200-fold. (TIFF 22173 kb)
