# Supplementary material for: Amyloid in biopsies of the gastrointestinal tract—a retrospective observational study on 542 patients
Source: Virchows Arch. 2016 Feb 25;468:569–77. doi: 10.1007/s00428-016-1916-y (PMC4856726; doi:10.1007/s00428-016-1916-y)
Supplement: Supplementary file 1 — (DOC 77 kb) [file 428_2016_1916_MOESM1_ESM.doc]

**Supplemental Table 1**: Mucosal to submucosal (“vertical”) distribution of amyloid in biopsies irrespective of biopsy site

| **Amyloid-type** | **Mucosa**  **interstitial**  **[n(%)]** | | | | **Mucosa**  **vascular**  **[n(%)]** | | | | **Muscularis mucosae**  **interstitial**  **[n(%)]** | | | | **Muscularis mucosae**  **vascular**  **[n(%)]** | | | | **Submucosa**  **interstitial**  **[n(%)]** | | | | **Submucosa**  **vascular**  **[n(%)]** | | | |
| --- | --- | --- | --- | --- | --- | --- | --- | --- | --- | --- | --- | --- | --- | --- | --- | --- | --- | --- | --- | --- | --- | --- | --- | --- |
|  | negativ | | positiv | | negativ | | positiv | | negativ | | positiv | | negativ | | positiv | | negativ | | positiv | | negativ | | positiv | |
| AL lambda | 73 | (21.2) | 271 | (78.8) | 174 | (50.6) | 170 | (49.4) | 15 | (4.4) | 329 | (95.6) | 112 | (32.6) | 232 | (67.4) | 9 | (2.6) | 333 | (97.4) | 23 | (6.7) | 318 | (93.3) |
| AL kappa | 10 | (10.8) | 83 | (89.2) | 30 | (32.3) | 63 | (67.7) | 2 | (2.2) | 89 | (97.8) | 19 | (20.9) | 72 | (79.1) | 1 | (1.1) | 88 | (98.9) | 2 | (2.2) | 87 | (97.8) |
| AL n.o.s. | 6 | (33.3) | 12 | (66.7) | 14 | (77.8) | 4 | (22.2) | 2 | (11.1) | 16 | (88.9) | 8 | (44.4) | 10 | (55.6) | 1 | (5.6) | 17 | (94.4) | 4 | (22.2) | 14 | (77.8) |
| ATTR | 90 | (86.5) | 14 | (13.5) | 95 | (91.3) | 9 | (8.7) | 63 | (61.2) | 40 | (38.8) | 42 | (40.8) | 61 | (59.2) | 53 | (52.0) | 49 | (48.0) | 1 | (1.0) | 101 | (99.0) |
| AA | 8 | (11.3) | 63 | (88.7) | 22 | (31.0) | 49 | (69.0) | 7 | (10.0) | 63 | (90.0) | 12 | (17.1) | 58 | (82.9) | 18 | (25.7) | 52 | (74.3) | 0 | (0.0) | 70 | (100.0) |
| AApoAI | 0 | (0.0) | 4 | (100.0) | 1 | (25.0) | 3 | (75.0) | 0 | (0.0) | 4 | (100.0) | 0 | (0.0) | 4 | (100.0) | 0 | (0.0) | 4 | (100.0) | 0 | (0.0) | 4 | (100.0) |
| ALys | 0 | (0.0) | 8 | (100.0) | 1 | (12.5) | 7 | (87.5) | 0 | (0.0) | 8 | (100.0) | 2 | (25.0) | 6 | (75.0) | 0 | (0.0) | 8 | (100.0) | 2 | (25.0) | 6 | (75.0) |
| Mixed | 0 | (0.0) | 3 | (100.0) | 1 | (33.3) | 2 | (66.7) | 0 | (0.0) | 3 | (100.0) | 1 | (33.3) | 2 | (66.7) | 0 | (0.0) | 3 | (100.0) | 0 | (0.0) | 3 | (100.0) |
| unclassified | 1 | (6.7) | 14 | (93.3) | 8 | (53.3) | 7 | (46.7) | 1 | (7.1) | 13 | (92.9) | 3 | (21.4) | 11 | (78.6) | 0 | (0.0) | 13 | (100.0) | 1 | (7.7) | 12 | (92.3) |
| Total | 188 | (28.5) | 472 | (71.5) | 346 | (52.4) | 314 | (47.6) | 90 | (13.7) | 565 | (86.3) | 199 | (30.4) | 456 | (69.6) | 82 | (12.6) | 567 | (87.4) | 33 | (5.1) | 615 | (94.9) |
