# Supplementary figures and images for: Amyloid in biopsies of the gastrointestinal tract—a retrospective observational study on 542 patients
Source: Virchows Arch. 2016 Feb 25;468:569–77. doi: 10.1007/s00428-016-1916-y (PMC4856726; doi:10.1007/s00428-016-1916-y)

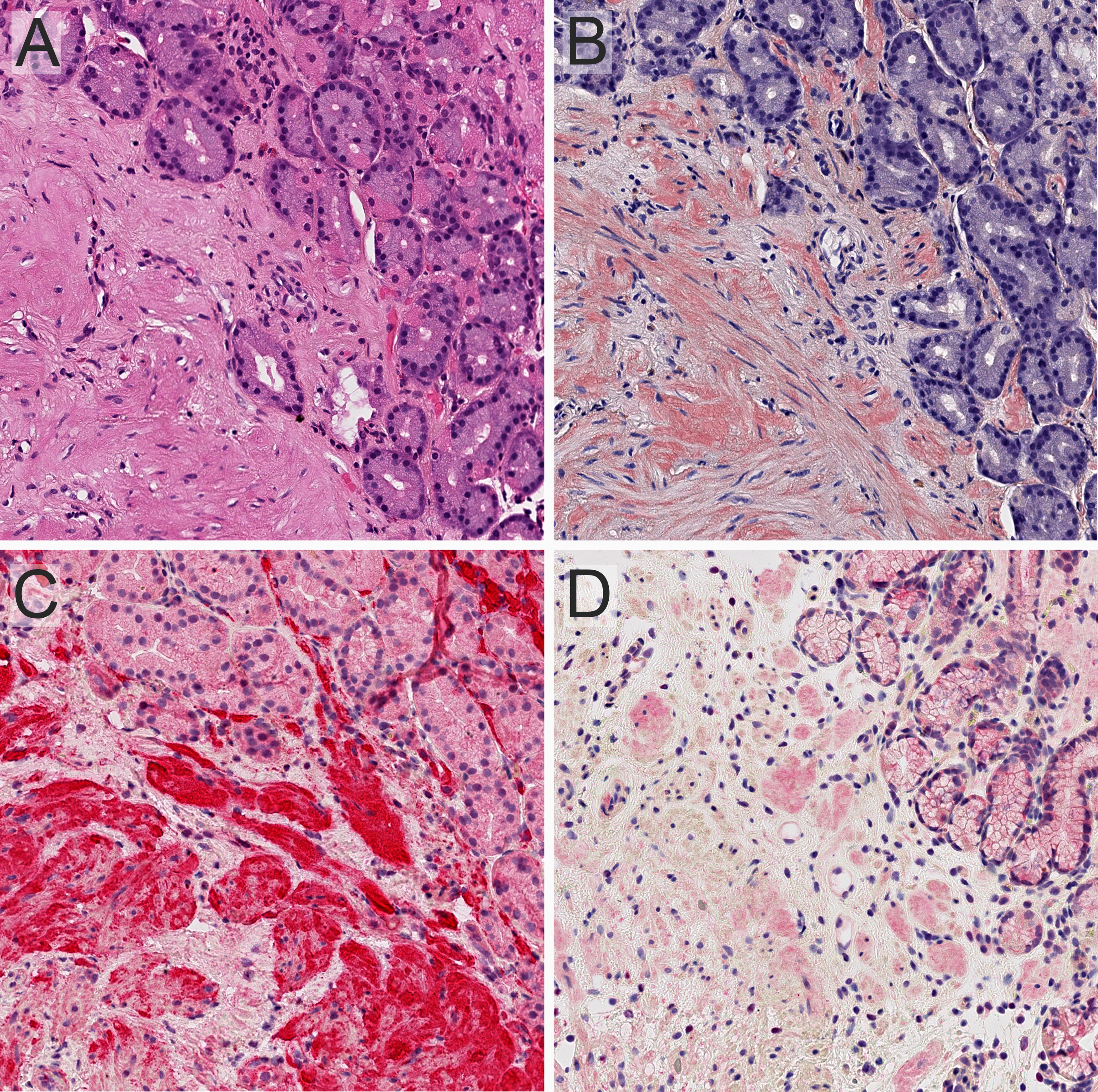

Supplement: Supplementary file 2 — ALλ amyloid in a gastric biopsy with typical involvement of the muscularis mucosae. On an H&E-stained section (A) a homogeneous eosinophilic material was found in the interstitium extending into the mucosa. Congo red stains the deposits (B) and shows yellow-green-orange birefringence (not shown). The amyloid deposits immunoreact with an antibody directed against lambda-light chain (C) but not with an antibody directed against transthyretin (D). Original magnifications 200-fold. (TIFF 22173 kb) [file 428_2016_1916_MOESM2_ESM.tif]
